# Use of Rapid Tranquillisation in Acute Inpatient Wards at Lanchester Road Hospital, Durham – Tees, Esk and Wear Valleys NHS Foundation Trust

**DOI:** 10.1192/bjo.2025.10590

**Published:** 2025-06-20

**Authors:** Pablo Fiks, Tom Hills, Archana Sreekumar, Alice Easton, Etido Effiong

**Affiliations:** 1Tees, Esk and Wear Valleys, Durham, United Kingdom; 2County Durham and Darlington NHS Trust, Durham, United Kingdom

## Abstract

**Aims:** To assess the current practice of rapid tranquillisation (RT) in acute inpatient psychiatric wards and compare this to trusts’ protocols.

To evaluate the adherence to NICE guidelines in the use of RT.

To provide recommendations for improving safety and adherence to local protocols in RT practices.

**Methods:** A retrospective audit of medical records from 2 acute inpatient wards during a three-month period in 2024.

Sample: We had a total of 237 administrations of RT, divided in between 16 patients. This total sample was then randomized, and we selected 99 RT administrations for data collection.

Data Collection: Review of patient records from a 3-month period (July–September 2024). We requested RT administration data from the trusts’ pharmacy team.

Key Indicators: We selected 18 key indicators which broadly belong to the following categories: Incident details, documentation, RT medication, patient characteristics and legal status.

Analysis: Descriptive and comparative analysis to identify trends, areas of non-compliance, and potential areas of improvement.

**Results:** Our data showed that in most cases (92%) there was a clear rationale recorded for using RT.

The majority of patients were under a section of the MHA (97%).

There was a record of oral medication offered prior to administration of RT in 68%.

Choice of RT medication was in line with the local TEWV RT guidelines in 75% of the cases. Lorazepam was the drug of choice in most of the cases.

In 80% of the cases, there was not adequate recording of post-RT observations, however we noted that in 57% of these cases there was a recorded refusal to have physical observations taken.

**Conclusion:** The audit revealed that while the use of rapid tranquillisation in acute inpatient wards is mostly in line with local protocols and NICE guidelines, however there are areas for improvement, particularly in documentation, post-RT monitoring, and adherence to RT protocols in terms of debriefing.

Recommendations for practice improvements include:

Ensure adherence to protocols to have consistent post-RT monitoring.

Regular audits to improve adherence to clinical guidelines.

By addressing these areas, we can improve patient safety, clinical outcomes, and staff confidence in the use of rapid tranquillisation in acute settings.

